# The impact of expectant management compared with intrauterine insemination with ovarian stimulation on quality of life and coital frequency in couples with unexplained subfertility

**DOI:** 10.1016/j.xfre.2025.06.001

**Published:** 2025-06-11

**Authors:** Jennifer A. Wessel, Monique H. Mochtar, Rik van Eekelen, Leoni A. Louwé, Eugenie M. Kaaijk, Mariëtte Goddijn, Madelon van Wely, Femke Mol

**Affiliations:** a Department of Obstetrics and Gynecology, Amsterdam UMC, University of Amsterdam, Amsterdam, the Netherlands; b Amsterdam Reproduction and Development research institute, Amsterdam, the Netherlands; c Department of Gynaecology, Leiden University Medical Center, Leiden, the Netherlands; d Department of Gynaecology, OLVG, Amsterdam, the Netherlands

**Keywords:** Unexplained subfertility, expectant management, intrauterine insemination, health-related quality of life, coital frequency

## Abstract

**Objective:**

To study the impact of 6 months of expectant management (EM) compared with 6 months of intrauterine insemination with ovarian stimulation (IUI-OS) on the health-related quality of life (HRQoL), anxiety and depression scores and the coital frequency in couples with unexplained subfertility and a poor prognosis for natural conception.

**Design:**

A study alongside the ExIUI trial, a multicentre randomized controlled trial.

**Subjects:**

Couples with unexplained subfertility and a poor prognosis for natural conception based on the score of Hunault.

**Intervention:**

Women in both groups were asked to fill in the Fertility Quality of Life (FertiQol) and Hospital Anxiety and Depression Scale (HADS) questionnaires at 3 moments and to keep a diary with their periods and dates of coitus.

**Main Outcome Measures:**

Health-related quality of life scores, anxiety and depression scores, and coital frequency.

**Results:**

Of the 178 women, 161 (90%) filled in at least one questionnaire. Women allocated to EM scored significantly lower on the relational domain compared with women allocated to IUI-OS (overall mean difference, –5.93; 95% confidence interval [CI], –10.64 to –1.22). We found no difference between the two groups in the social domain (overall mean difference, –4.72; 95% CI, –9.88 to 0.45). No difference between the two was found in anxiety (overall mean difference, –0.20; 95% CI, –1.44 to 1.04) or depression scores (overall mean difference, 0.22; 95%CI, –0.86 to 1.30). Among all women allocated to either EM or IUI-OS, anxiety and depression scores increased over time. Seventy-nine (44%) out of 178 women filled in at least one month of their diary and reported on a total of 497 cycles and registered 2,026 dates of coitus. The median coital frequency per cycle was 4 (interquartile range [IQR]: 1–7) in the EM group and 3 (IQR: 0–7) in the IUI-OS group. The median coital frequency in the fertile window per cycle was 2 (IQR: 0–3) in EM and 1 (IQR: 0–3) in IUI-OS, which was a statistically significant difference.

**Conclusion:**

Compared with IUI-OS, 6 months of EM resulted in a lower HRQoL on the relational domain, comparable anxiety and depression scores and higher coital frequency in the fertile window.

**Clinical Trial Registration Number:**

Dutch Trial register NL5455 (NTR5599).

When couples are experiencing difficulties conceiving, it induces significant levels of stress and emotional distress. Continuing to try to conceive at home can feel such as wasting time, and couples experience a lack of confidence in natural conception (1). At the same time, starting fertility treatment can offer hope, but the procedures and failure can also be stressful (2, 3).

Decreased quality of life and symptoms of anxiety and depression may hinder women from initiating and adhering to fertility treatments, leading to higher rates of discontinuation (4). Therefore, it is important to assess the couple's well-being at the intake and to determine whether initiating fertility treatment affects women’s health-related quality of life (HRQoL) or anxiety or depression scores.

It is known that the prevalence of anxiety and depression disorders is increased in couples with subfertility compared with fertile couples and even higher after failed treatments (5). It is not without reason that clinical guidelines recommend having mental health professionals as part of the team or maintaining close contact with external professionals (6, 7). It is our hypothesis that starting intrauterine insemination with ovarian stimulation (IUI-OS) results in less change in HRQoL or anxiety and depression scores than 6 months of expectant management (EM).

In addition to biological factors such as ovulation and sperm quality, behavioral aspects such as coital frequency also play a critical role in conception. Coital frequency is influenced by multiple factors, for example, age, working status and exercise, but it is unknown whether the coital frequency is affected by starting fertility treatment (8). It is known that the sexuality of couples could change as a result of the unfulfilled child's wish (9). The wish for a child can become an obsession, impairing sexual pleasure and making it feel like an obligation or failure (10, 11). Our hypothesis is that couples with unexplained subfertility who were allocated to the EM group have a lower coital frequency compared with the IUI-OS group, and that this may partly explain the effect of IUI-OS on pregnancy chances.

In this study, we looked at the impact of 6 months EM compared with 6 months IUI-OS on HRQoL, anxiety and depression scores and on coital frequency in couples with unexplained subfertility and a poor prognosis for natural conception. By comparing these two approaches, the study aimed to provide better insight into how fertility treatments affect quality of life, mental health, and sexual behavior.

## Materials and methods

### Study design and participants

We performed this study alongside the ExIUI study, a randomized controlled trial comparing EM with IUI-OS in couples with unexplained subfertility and a poor prognosis for natural conception (12). This study was approved by the Medical Ethics Committee of AMC Amsterdam (METC 2016_133, NL 57383.018.16) and by the boards of all participating hospitals. A total of 14 hospitals included couples in the study. All participants provided written informed consent before randomization. The study was registered at the Dutch Trial Register NL5455 (NTR5599).

Heterosexual couples who were diagnosed with unexplained subfertility and a poor prognosis for natural conception were eligible. A poor prognosis was defined as (1) a Hunault score <30% (13), (2) unexplained subfertility and age between 38–43 years or (3) when couples with an initial Hunault score >30% had returned after 6 months of EM. The model of Hunault is an externally validated tool that includes female age, duration of subfertility, sperm motility, history of previous pregnancy, and referral status. The model combines these variables to predict the likelihood of couples conceiving naturally in the next 12 months (13).

We excluded couples with a previous fertility treatment in the current infertility episode, using donor sperm, with a medical contraindication for pregnancy or with sexual problems interfering with the chance of natural conception.

### Procedures

After written informed consent, couples were randomly assigned to EM, which means no intervention or counseling, or IUI-OS for 6 months. At the start of the study, after randomization (T0), after 3 months (T1), and after 6 months (T2) the women who could read Dutch were asked to fill in two questionnaires: the Dutch version of the Fertility Quality of Life (FertiQol) to assess HRQoL and the Dutch version of the Hospital Anxiety and Depression Scale (HADS) to assess anxiety and depression symptoms (Supplemental Fig. 1, available online). Women were asked to keep a diary with their menstruation dates and the dates they had coitus during the study period (Supplemental Fig. 2, available online).

### Outcomes

#### Health-related quality of life

The FertiQol questionnaire is a specific and reliable tool for the assessment of quality of life. Officially, it comprises four domains: mind-body, emotional, relational, and social. We have opted to include only the relational and social domains because these components are most applicable. Each domain contains six questions on a five-point scale. Scores can range from 0–100, and a higher score indicates a better quality of life (3, 14). The FertiQol questionnaire has undergone validation in multiple countries, showing good overall psychometric properties and remains validated even if only part of it is used (www.fertiqol.org) (3).

#### Anxiety and depression scores

The HADS questionnaire is a reliable tool to detect anxiety and depression disorders. The questionnaire contains 14 questions, seven for anxiety and seven for depression, all on a four-point scale. The score ranges from 0–21. A HADS score of eight or higher signifies the presence of anxiety and/or depression symptoms. The higher the score, the more severe the symptoms (15, 16). In 1997, the Dutch version of the HADS questionnaire was validated in different groups of patients. The findings of that study align with the validation of the original HADS, indicating stable dimensional structure and reliability of the scales across various medical settings and age groups (15).

#### Coital frequency and timing

The coital frequency was calculated per cycle and during the fertile window. We defined the fertile window as 7 days before and 2 days after the estimated ovulation date for all cycles. We estimated the day of ovulation based on the date of the next menstrual period minus 14 days in the EM group.

### Statistical analysis

The analysis was based on intention to treat. The FertiQol and HADS scores were expressed as mean ± standard deviation (SD) and mean differences between the time frames. We used generalized linear mixed models corrected for baseline value to assess differences between EM and IUI-OS over time. We calculated overall mean differences with 95% confidence intervals (CIs) and tested for interaction between treatment and time. For coital frequency, we used the Mann-Whitney *U* test for differences in frequency and timing and logistic regression for the estimated association with live birth, adjusting for cycle number and treatment allocation. Differences were expressed as adjusted odds ratio (OR) with 95% CI. To assess whether nonresponder bias may have affected results, we compared the baseline outcomes between women who filled in a diary and those who did not.

The analyses were performed using SPSS software (version 28.0; IBM Corp., Armonk, New York).

## Results

Between October 2016 and September 2020, 178 couples were randomized between EM and IUI-OS for the initial ExIUI randomized controlled trial (RCT). Of 178 included couples, 92 were allocated to EM and 86 to IUI-OS. Couples allocated to EM had a lower live birth rate (12/92 [13%]) than couples allocated to IUI-OS (28/86 [33%], resulting in a relative risk of 0.40; 90% CI, 0.24–0.67) (12).

Supplemental Table 1 (available online) shows the baseline characteristics for three groups the whole study group from the initial ExIUI RCT; women who completed a questionnaire for this study; and women who filled in at least one month of a diary for this study. The mean female age was 34 years in both groups, and the mean duration of subfertility was 22 months in the EM group and 21 months in the IUI-OS group (12). Baseline characteristics did not differ between the three groups.

The FertiQol questionnaire was filled in by 85% of patients at baseline (151/178), 58% at 3 months (103/178) and 61% at 6 months (108/178). Eighty-eight percent filled in the HADS questionnaire at baseline (156/178), 63% at 3 months (112/178) and 66% at 6 months (118/178). Of the women, 44% (79/178) filled out at least one monthly diary on coital frequency and menstruation dates, 44 allocated to EM and 35 allocated to IUI-OS.

### Health-related quality of life: relational and social domain

Mean scores of the FertiQol questionnaires and the overall mean differences between EM and IUI-OS are presented in Table 1. In the last three columns of this table, the *P* values of the treatment effect, time effect, and the interaction between those two are shown.

**Table 1:** Mean scores for FertiQol and HADS at three moments in time and the overall adjusted difference between the intervention groups. *P* values for the treatment, time and time x treatment effect between both groups.

	Mean score (SD) before randomization		Mean score (SD) at 3 months after randomization		Mean score (SD) at 6 months after randomization		Overall difference adjusted for baseline	Treatment effect p-value	Time effect p-value	Time x treatment p-value
FertiQol	EM	IUI-OS	EM	IUI-OS group	EM	IUI-OS				
Relational domain	79 (12)	80 (12)	71 (17)	80 (14)	71 (16)	78 (17)	–5.93 (–10.64 to –1.22)	.014	.44	<.001
Social domain	77 (14)	76 (17)	68 (21)	74 (18)	68 (17)	69 (21)	–4.72 (–9.88 to 0.45)	.07	.05	.06
HADS	EM	IUI-OS	EM	IUI-OS group	EM	IUI-OS				
Anxiety scale	4.7 (3.3)	5.0 (3.4)	6.5 (4.6)	5.6 (4.2)	5.5 (3.7)	6.3 (4.3)	–0.20 (–1.44 to 1.04)	.75	.13	.24
Depression scale	2.4 (2.5)	2.7 (2.4)	4.0 (3.57)	3.1 (3.5)	3.9 (3.8)	4.1 (3.9)	0.22 (–0.86 to 1.30)	.69	<.001	.10

*Note:* The FertiQol subscale scores were transformed into a 0–100 scale, with higher scores meaning higher quality of life.

The HADS subscale scores indicate a total score, with higher scores (above 8) indicating a severe situation for anxiety or depression. EM = expectant management, IUI-OS = intrauterine insemination with ovarian stimulation.

**Figure 1:**
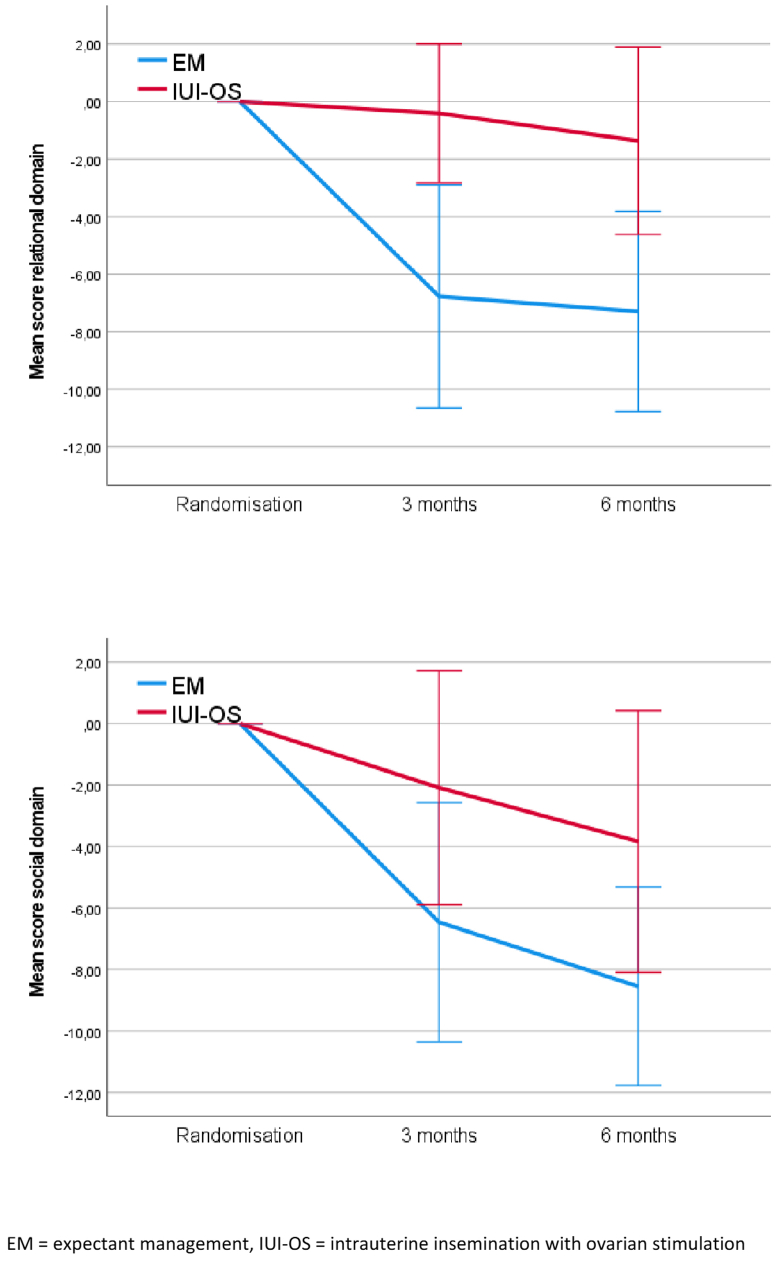
FertiQol mean scores for relational and social domain with baseline as covariable. EM = expectant management, IUI-OS = intrauterine insemination with ovarian stimulation.

In women allocated to EM, FertiQol scores in the relational domain decreased over time with no decrease in the IUI-OS group, resulting in a significant treatment effect (overall mean difference –5.93; 95% CI, –10.64 to –1.22; *P*=.014) and a significant interaction between treatment and time (*P*<.001). Similarly, in women allocated to EM the social domain scores decreased over time with a smaller decrease in the IUI-OS group, although not resulting in a significant difference between EM and IUI-OS (overall mean difference, –4.72; 95% CI, –9.88 to 0.45; *P*=.07, time effect *P*=.05, interaction *P*=.06). (Table 1 and Fig. 1).

### Anxiety and depression scores

**Figure 2:**
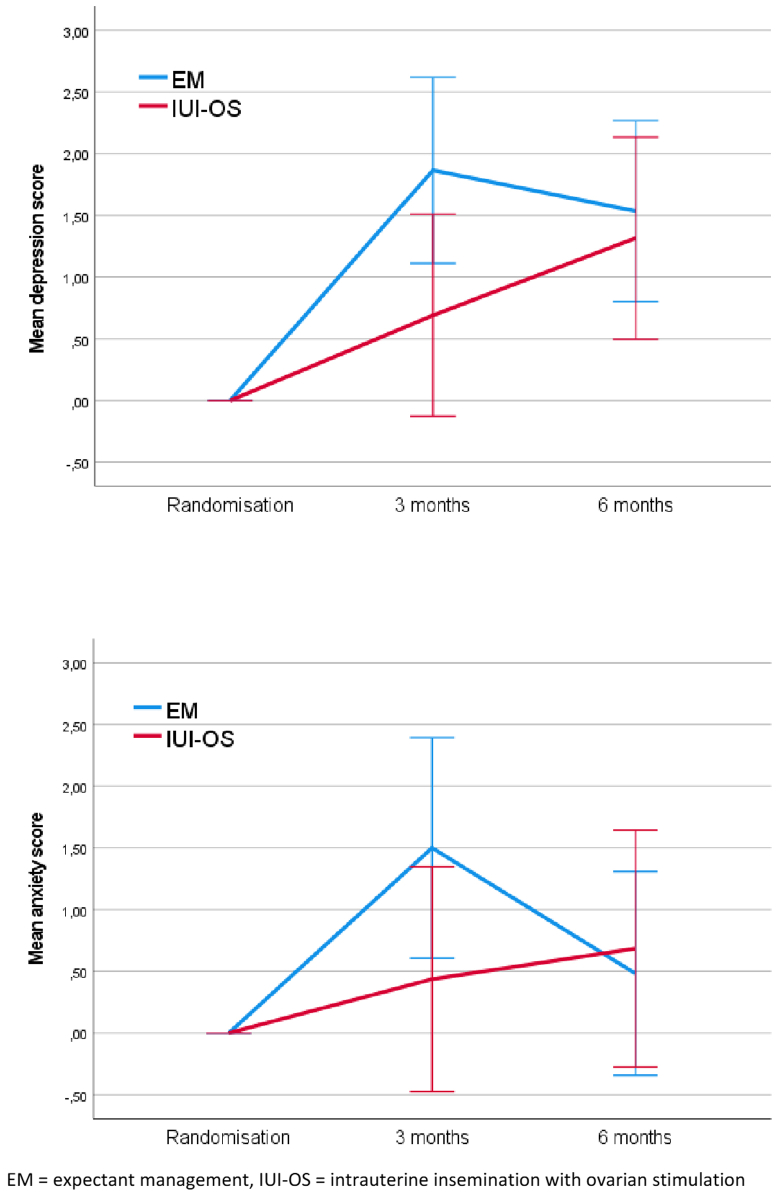
HADS mean scores for depression and social domain with baseline as covariable. EM = expectant management, IUI-OS = intrauterine insemination with ovarian stimulation.

Table 1 also presents the mean scores and the overall mean differences of the HADS questionnaires. In both anxiety and depression, the EM group initially shows a faster increase in scores compared with the IUI-OS group, but both groups reach approximately the same score after 6 months. No difference between the intervention groups was found in anxiety scores (overall mean difference, –0.20; 95% CI, –1.44 to 1.04) and depression scores (overall mean difference, 0.22; 95% CI, –0.86 to 1.30) (Table 1 and Fig. 2). We observed a significant difference in depression scores over time. As time progresses, these scores tend to increase in both groups (*P*≤.001). There was no interaction between treatment and time on anxiety or depression scores.

The proportion of women with anxiety symptoms (HADS score eight or higher) increased from 14% (11/77) at baseline to 26% (16/61) at 6 months in the EM group vs. 24% (19/79) to 33% (19/57) in the IUI-OS group (*P*=.46). The prevalence of depressive symptoms (score of eight or higher) increased in both groups, with a larger increase in the IUI-OS group, although this difference was not statistically significant. In the EM group, the prevalence increased from 5.2% (4/77) at baseline to 15% (9/61) at 6 months, whereas in the IUI-OS group it increased from 3.8% (3/79) to 21% (12/57) (*P*=.48) (Supplemental Fig. 3, available online).

### Coital frequency and timing

The 79 women (79/178, 44%) who filled in at least one month of the diary reported 497 cycles and 2,026 dates of coitus. Per cycle, the average coital frequency was 4.1. The median coital frequency was 4 (interquartile range [IQR]: 1–7) during EM and 3 (IQR: 0–7) during IUI-OS. The median coital frequency in the fertile window was 2 (IQR: 0–3) during EM and 1 (IQR: 0–3) during IUI-OS (*P*<.01) (Table 2). We have not included the IUI itself in this table.

**Table 2:** Coital frequency per cycle in total and in the fertile window.

Outcome	EM (n = 44)	IUI-OS (n = 35)	*P* value
Median coital frequency total	4 (IQR 1–7)	3 (IQR 0–7)	.08
Median coital frequency in fertile window	2 (IQR 03)	1 (IQR 0–3)	<.01

*Note:* The fertile window is defined as 7 days before and 2 days after the estimated ovulation date. EM = expectant management, IQR = interquartile range, IUI-OS = intrauterine insemination with ovarian stimulation.

The adjusted odds ratio for coital frequency was 0.93 (95% CI, 0.79–1.10). The adjusted OR for coitus in the fertile window was 0.96 (95% CI, 0.66–1.41). The OR for allocated treatment was unaffected by these adjustments for coital frequency or timing, both in terms of a simple adjustment or an interaction between coital frequency or timing and treatment.

Women who did or did not complete a diary did not show a statistically significant difference in baseline scores regarding the relational domain or anxiety and depression scores. Compared with women who did not complete a diary, women who completed a diary had a lower score in the social domain (mean difference, –5.02 [95% CI, –10.02 to –0.03]) (Supplemental Table 2 and Fig. 4, available online).

## Discussion

Alongside an RCT (ExIUI trial), we investigated HRQoL, anxiety and depression scores and coital frequency in couples with unexplained subfertility undergoing 6 months of EM or IUI-OS. Overall, we did not notice major differences in the HRQoL between EM and IUI-OS, except in the relational domain. This domain was assessed by six questions, and we found that women allocated to EM scored especially lower on the question of whether they experienced difficulties in communicating about their feelings with their partner compared with women allocated to IUI-OS. We believe that the longer the subfertility persists, the more frustrating it becomes, potentially leading to communication problems and a shift in the relationship. The absence of a decline in HRQoL in the IUI-OS group in our study may be attributed to couples feeling more hopeful on initiating treatment. However, it is also evident that couples may disagree over unequal participation in examinations and treatments (2, 17). Interestingly, this disagreement does not reflect in the scores on the relational domain in our study.

No difference was found in anxiety and depression scores between the two groups. Scores increased over the 6-month period in both groups, only the increase in depression scores was statistically significant. This can likely be explained by the prolonged duration of subfertility. It seems the group allocated to EM quickly worsens after randomization, whereafter both groups normalize to the same values of anxiety and depression at 6 months. This potential interaction between treatment and time on anxiety and depression scores was not found to be statistically significant; however, this could be because of the relatively low power of the study.

Couples allocated to EM had a higher coital frequency in the fertile window than those allocated to IUI-OS. A plausible explanation is that partners were advised abstinence for several days in anticipation of IUI, with the protocol recommending 2–5 days. In the study data, it is unclear whether this advice was explicitly communicated to the participants or if the men adhered to it. In addition, we have chosen not to include the IUI as coitus in the table. If we had done so, the frequency in both groups would be identical, and there would be no significant difference between the two groups. We found an average coital frequency of four times/cycle during EM, which is higher than the average of the general Dutch population of three times a month as found in a national survey of sexual health in 2017 in men and women aged between 25 and 39 years (18). We do not have specific details about what was discussed during the consultations with the physician, particularly regarding the benefits of regular coitus for increasing the chances of pregnancy or explanations about the fertile window. It is unclear whether this information was provided equally to both groups. However, a study by Martins et al. (19) found that providing education about the fertile window did not significantly impact sexual intercourse patterns compared with a control group that received no such education.

A previous study showed a possible association with anxiety or depression and coital frequency in couples who were trying to conceive without a history of infertility (8). We would have liked to assess this combination of anxiety and/or depression and the coital frequency of couples; however, in our study, the number of questionnaires and diaries was too small to perform these analyses. In addition, we recommend further research on questioning the partners about their HRQoL and coital frequency. In recent years, there is growing evidence that subfertility also affects men’s quality of life, leading to increased anxiety and depression because of factors such as self-blame or shame (5). Furthermore, the impact of quality of life and anxiety and depression on patient preference is understudied and needs to be taken into account in future patient preference studies (20).

A strength of our study is that we used validated questionnaires to evaluate HRQoL and anxiety and depression scores, and as far as we know, to our knowledge, this is the first study that asked women to keep a diary during both EM and IUI-OS.

The main limitation of our study is that the initial study was halted prematurely because of slow recruitment, lack of funding and study fatigue, and therefore did not reach the planned sample size of 1,091 couples. As a result, we were unable to perform reliable analyses of HRQoL scores when split by whether patients conceived or not during the study period, because of the small overall number of patients. Furthermore, the diaries with coital frequency and menstruation dates were returned by only 44% of the women, which could have induced selection bias. We did not ask the women to track their ovulation, so we estimated the day of ovulation based on the date of the next menstrual period in the EM group. In addition, it could be that women did not keep the diary in the cycle they conceived, leading to biased estimates for coital frequency and timing.

A second limitation of our study may be that we included only the relation and social domains of the FertiQol questionnaires because we found these two components most suitable. Additionally, by keeping the questionnaire shorter, we aimed to increase the likelihood of more women completing it. As with questionnaires, there is always the risk of response bias, and it is possible that women experiencing less mental distress may complete the questionnaire more promptly than those experiencing greater mental distress.

A third limitation is that we do not have information about the timing of questionnaire completion. This could be relevant as the timing of questionnaire completion, for instance at the start of the menstrual cycle or at the day of the insemination, could have impact on women’s mental health, there is no reason to believe that this was systematically different between groups.

Our baseline scores closely resemble the baseline score found in an RCT in couples with unexplained subfertility (21). Our depression and anxiety scores are lower than observed in the literature; this could be because of a shorter duration of infertility in our study population (22).

## Conclusion

To conclude, starting IUI-OS was not associated with an improvement in the social domain of HRQoL or in anxiety and depression scores in women with unexplained subfertility and a poor prognosis for natural conception. However, in the relational domain, initiating IUI-OS was associated with a decreased decline in relationship quality compared with EM. Practitioners should be aware of the increasing risk of symptoms of anxiety and depression over treatment time. An important finding of our study was that the mean coital frequency was not affected by EM, and therefore, we may conclude, with caution, that the lower pregnancy chance in the EM group compared with IUI-OS was not because of lack of coitus. Because our study sample was small, we need further studies on the mental health of couples with unexplained subfertility. We also need further studies to explore how we can identify and support these couples with unexplained subfertility and poor prognosis for natural conception, with anxiety and depression symptoms.

## Data Sharing Statement

The data underlying this article will be shared on reasonable request to the corresponding author.

## Declaration Of Interests

J.A.W. has nothing to disclose. M.H.M. has nothing to disclose. R.E. has nothing to disclose. L.A.L. has nothing to disclose. E.M.K. has nothing to disclose. M.G. has nothing to disclose. M.W. has nothing to disclose. F.M. has nothing to disclose.
