# Supplementary material for: The impact of expectant management compared with intrauterine insemination with ovarian stimulation on quality of life and coital frequency in couples with unexplained subfertility
Source: F S Rep. 2025 Jun 11;6(3):374–80. doi: 10.1016/j.xfre.2025.06.001 (PMC12496428; doi:10.1016/j.xfre.2025.06.001)
Supplement: Supplementary Figure S1 [file mmc1.pdf]

1. Hospital Anxiety and Depression Scale (HADS)

Het is bekend dat emoties bij de meeste ziektes een belangrijke rol kunnen spelen.
Deze vragenlijst dient als hulpmiddel om te weten te komen hoe u zich voelt. Lees iedere vraag en vink het antwoord aan dat het beste weergeeft hoe u zich **gedurende de laatste week** gevoeld heeft.

Denk niet te lang na over uw antwoord. Uw eerste reactie op elke vraag is waarschijnlijk betrouwbaarder dan een lang doordacht antwoord.

1. **Ik voel me gespannen**:

- ☐ Meestal
- ☐ Vaak
- ☐ Af en toe, soms
- ☐ Helemaal niet

2. **Ik geniet nog steeds van de dingen waar ik vroeger van genoot**:

- ☐ Zeker zo veel
- ☐ Niet zo veel als vroeger
- ☐ Weinig
- ☐ Haast helemaal niet

3. **Ik krijg een soort angstgevoel alsof er elk moment iets vreselijks zal gebeuren**:

- ☐ Heel zeker en vrij erg
- ☐ Ja, maar niet zo erg
- ☐ Een beetje, maar ik maak me er geen zorgen over
- ☐ Helemaal niet

4. **Ik kan lachen en de dingen van de vrolijke kant zien**:

- ☐ Net zoveel als vroeger
- ☐ Niet zo goed als vroeger
- ☐ Beslist niet zoveel als vroeger
- ☐ Helemaal niet

5. **Ik maak me vaak ongerust**:

- ☐ Heel erg vaak
- ☐ Vaak
- ☐ Af en toe maar niet te vaak
- ☐ Alleen soms

6. Ik voel me opgewekt

- ☐ Helemaal niet
- ☐ Niet vaak
- ☐ Soms
- ☐ Meestal

7. **Ik kan rustig zitten en me ontspannen:**

- ☐ Zeker
- ☐ Meestal
- ☐ Niet vaak
- ☐ Helemaal niet

8. **Ik voel me alsof alles moeizamer gaat:**

- ☐ Bijna altijd
- ☐ Heel vaak
- ☐ Soms
- ☐ Helemaal niet

9. **Ik krijg een soort benauwd, gespannen gevoel in mijn maag:**

- ☐ Helemaal niet
- ☐ Soms
- ☐ Vrij vaak
- ☐ Heel vaak

10. **Ik heb geen interesse meer in mijn uiterlijk:**

- ☐ Zeker
- ☐ Niet meer zoveel als ik zou moeten
- ☐ Waarschijnlijk niet zoveel
- ☐ Evenveel interesse als vroeger

11. **Ik voel me rusteloos en voel dat ik iets te doen moet hebben:**

- ☐ Heel erg
- ☐ Tamelijk veel
- ☐ Niet erg veel
- ☐ Helemaal niet

12. **Ik verheug me van tevoren al op dingen:**

- ☐ Net zoveel als vroeger
- ☐ Een beetje minder dan vroeger
- ☐ Zeker minder dan vroeger
- ☐ Bijna nooit

13. **Ik krijg plotseling gevoelens van panische angst:**

- ☐ Zeer vaak
- ☐ Tamelijk vaak
- ☐ Niet erg vaak
- ☐ Helemaal niet

14. **Ik kan van een goed boek genieten, of van een radio- of televisieprogramma:**

- ☐ Vaak
- ☐ Soms
- ☐ Niet vaak
- ☐ Heel zelden

FertiQoL International

Fertiliteit Kwaliteit van Leven Vragenlijst (2008)

Kies bij elke vraag het antwoord dat het beste aangeeft wat u denkt en voelt (plaats een vinkje in het bijbehorende hokje). Uw antwoorden moeten weergeven wat u op dit moment denkt en voelt. Sommigen vragen kunnen gaan over uw privé-leven. Deze vragen zijn echter nodig om een compleet beeld te krijgen van uw leven.

Vul de items die met een sterretje (*) zijn gemarkeerd alleen in als u een partner heeft.

	Kies bij elke vraag het antwoord dat het beste weergeeft hoe u zich op dit moment voelt	Zeer slecht	Slecht	Niet goed, niet slecht	Goed	Zeer goed
A	Hoe is uw gezondheid volgens u?	☐	☐	☐	☐	☐
	Kies bij elke vraag het antwoord dat het beste weergeeft hoe u zich op dit moment voelt	Zeer ontevreden	On-tevreden	Niet tevreden, niet ontevreden	Tevreden	Zeer tevreden
B	Bent u tevreden met de kwaliteit van uw leven?	☐	☐	☐	☐	☐
	Kies bij elke vraag het antwoord dat het beste weergeeft hoe u zich op dit moment voelt	Absoluut	In hoge mate	In zekere mate	Niet zo erg	Helemaal niet
	ga door naar vraag Q5 en verder6			☐	☐	☐
		☐			☐	
		☐			☐	☐
				☐		
	Kies bij elke vraag het antwoord dat het beste weergeeft hoe u zich op dit moment voelt	Zeer ontevreden	On-tevreden	Niet tevreden, niet ontevreden	Tevreden	Zeer tevreden
Q5	Bent u tevreden met de steun die u krijgt van vriend(inn)en met betrekking tot uw kinderwens?	☐	☐	☐	☐	☐
*Q6	Bent u tevreden met uw seksuele relatie ondanks dat u nog niet zwanger bent?	☐	☐	☐	☐	☐
	Kies bij elke vraag het antwoord dat het beste weergeeft hoe u zich op dit moment voelt	Altijd	Zeer vaak	Redelijk vaak	Zelden	Nooit
	ga door naar vraag Q10 en verder					
Q10	Bent u in sociaal opzicht geïsoleerd omdat u nog niet zwanger bent?	☐	☐	☐	☐	☐
*Q11	Gaan uw partner en u teder en liefhebbend met elkaar om ondanks het feit dat u nog niet zwanger bent?	☐	☐	☐	☐	☐
	ga door naar vraag Q13 en verder					
Q13	Voelt u zich vanwege uw kinderwens ongemakkelijk bij sociale gelegenheden als vakanties en festiviteiten?	☐	☐	☐	☐	☐
Q14	Denkt u dat uw familie kan begrijpen wat u doormaakt?	☐	☐	☐	☐	☐
	Kies bij elke vraag het antwoord dat het beste weergeeft hoe u zich op dit moment voelt	In extreem hoge mate	In hoge mate	In zekere mate	Een beetje	Helemaal niet
*Q15	Heeft het uw kinderwens de band met uw partner versterkt?	☐	☐	☐	☐	☐
	ga door naar vraag Q17					
Q17	Voelt u zich, omdat u nog niet zwanger bent, minderwaardig ten opzichte van mensen met kinderen?	☐	☐	☐	☐	☐
	ga door naar vraag Q19-Q22					
*Q19	Heeft het feit dat u nog niet zwanger bent een negatieve invloed gehad op uw relatie?	☐	☐	☐	☐	☐
*Q20	Vindt u het moeilijk om met uw partner over uw gevoelens met betrekking tot uw kinderwens te spreken?	☐	☐	☐	☐	☐
*Q21	Bent u tevreden met uw relatie ondanks het feit dat u nog niet zwanger bent?	☐	☐	☐	☐	☐
Q22	Voelt u sociale druk om (meer) kinderen te krijgen?	☐	☐	☐	☐	☐
	ga alleen door naar de laatste pagina als u een vruchtbaarheidsbehandeling begonnen bent.					

© European Society of Human Reproduction & Embryology and American Society of Reproductive Medicine

Supplementary Figure S1. FertiQol and HADS questionnaires in Dutch.
