# Supplementary material for: The impact of expectant management compared with intrauterine insemination with ovarian stimulation on quality of life and coital frequency in couples with unexplained subfertility
Source: F S Rep. 2025 Jun 11;6(3):374–80. doi: 10.1016/j.xfre.2025.06.001 (PMC12496428; doi:10.1016/j.xfre.2025.06.001)
Supplement: Supplementary Figure S2 [file mmc2.pdf]

Week 1	1	2	3	4	5	6	7
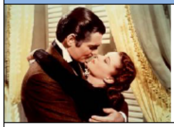							
Menstruatie							
**Week 2**	**1**	**2**	**3**	**4**	**5**	**6**	**7**
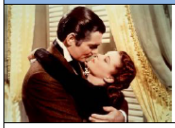							
Menstruatie							

nvog consortium 2.0

Supplementary Figure S2. Example of the diary that women were given to complete.
First line: month and dates, 1 = cycle day 1.
Second line: coitus
Third line: period
