# Supplementary material for: The impact of expectant management compared with intrauterine insemination with ovarian stimulation on quality of life and coital frequency in couples with unexplained subfertility
Source: F S Rep. 2025 Jun 11;6(3):374–80. doi: 10.1016/j.xfre.2025.06.001 (PMC12496428; doi:10.1016/j.xfre.2025.06.001)
Supplement: Supplementary Figure S3 [file mmc3.pdf]

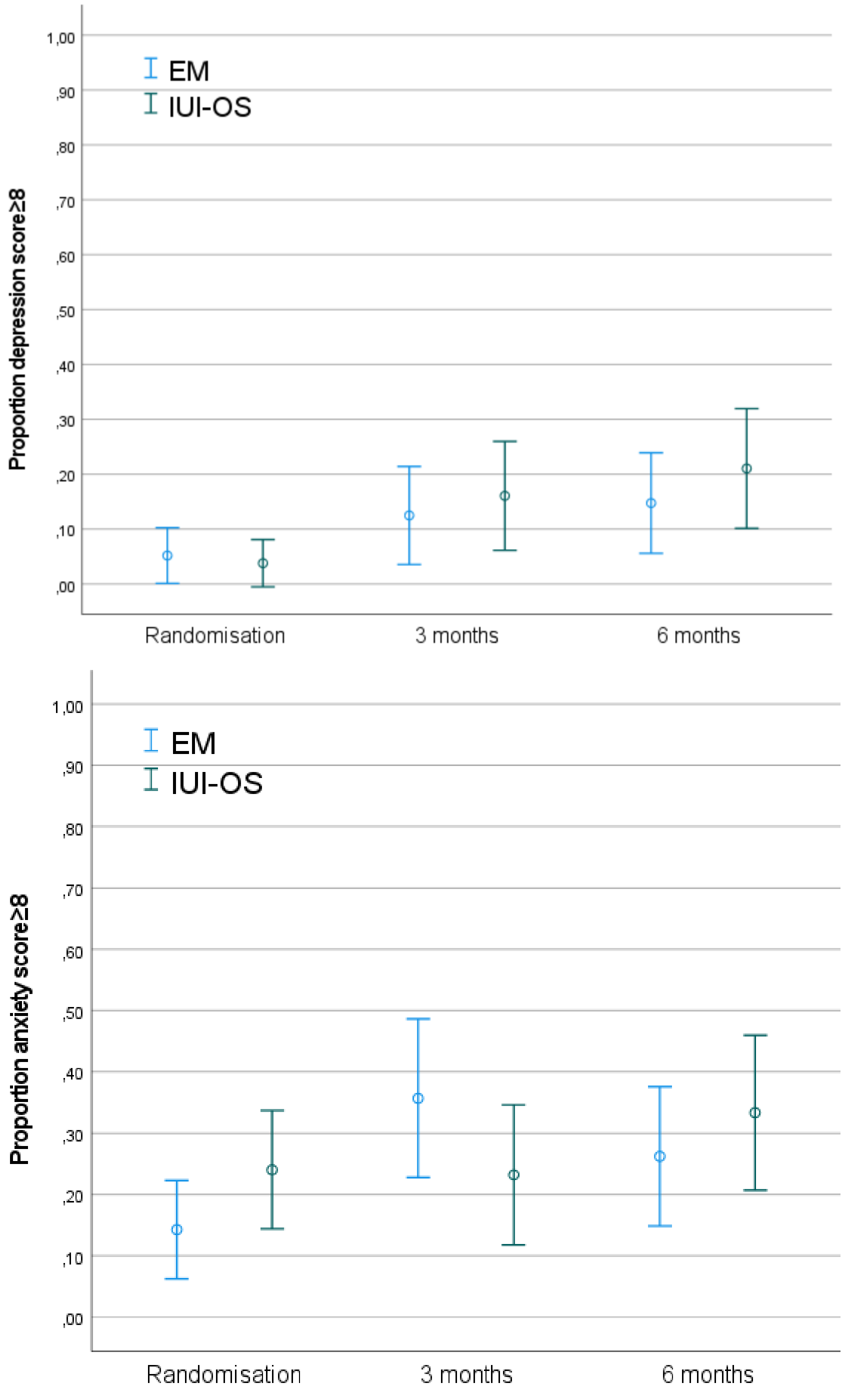

EM = expectant management, IUI-OS = intrauterine insemination with ovarian stimulation

Supplementary Figure S3. Proportion of a depression and anxiety score ≥ 8 at the three different time moments
