# Supplementary material for: The impact of expectant management compared with intrauterine insemination with ovarian stimulation on quality of life and coital frequency in couples with unexplained subfertility
Source: F S Rep. 2025 Jun 11;6(3):374–80. doi: 10.1016/j.xfre.2025.06.001 (PMC12496428; doi:10.1016/j.xfre.2025.06.001)
Supplement: Supplementary Figure S4 [file mmc4.pdf]

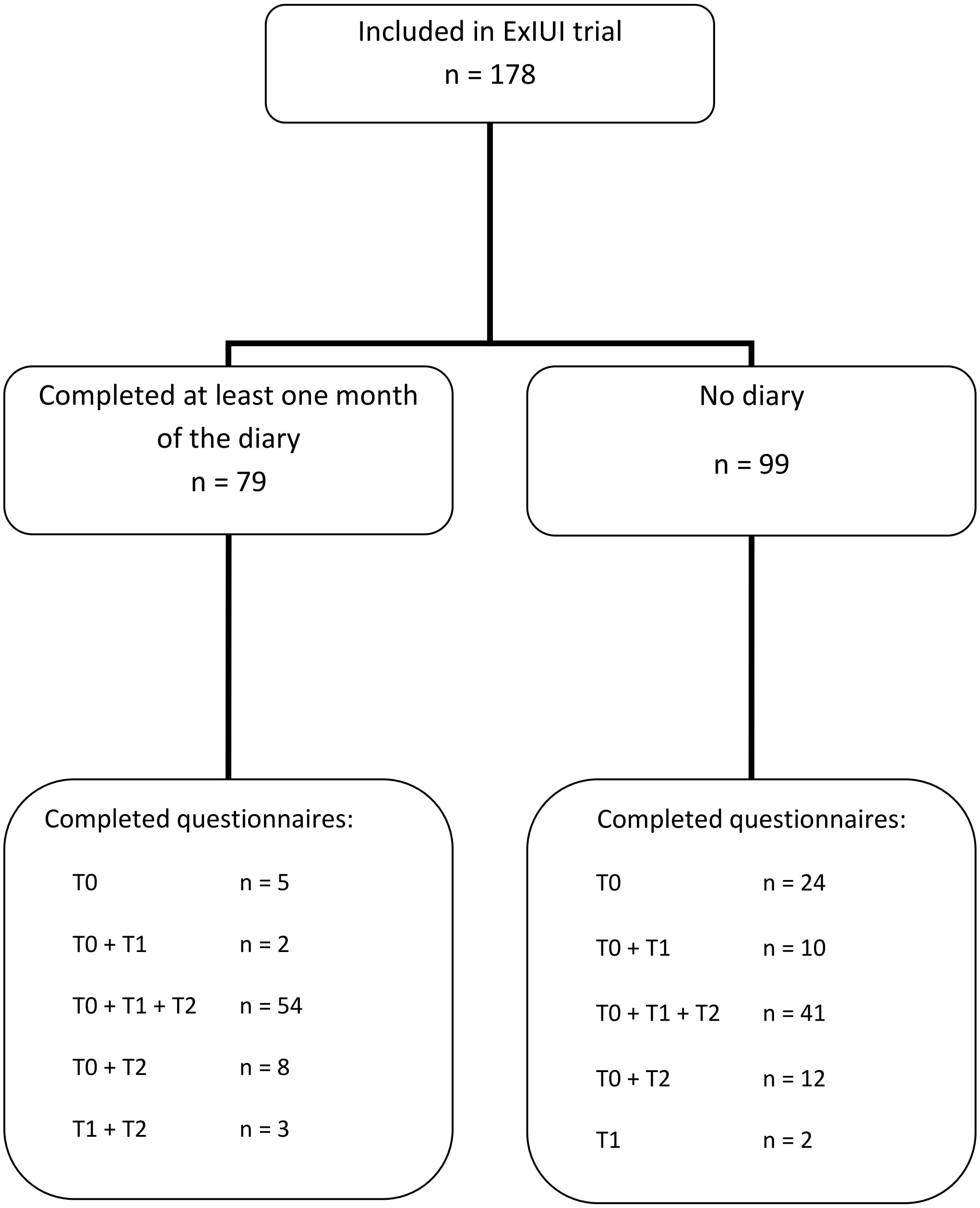

T0 = randomisation
T1 = 3 months after randomisation
T2 = 6 months after randomisation

Supplementary figure S4. Flow diagram depicting whether women kept a diary and subsequently completed the questionnaires.
