# Supplementary material for: The impact of expectant management compared with intrauterine insemination with ovarian stimulation on quality of life and coital frequency in couples with unexplained subfertility
Source: F S Rep. 2025 Jun 11;6(3):374–80. doi: 10.1016/j.xfre.2025.06.001 (PMC12496428; doi:10.1016/j.xfre.2025.06.001)
Supplement: Supplementary Table 1 [file mmc5.docx]

| Outcome | n | Expectant management | n | IUI-OS | p-value |
| --- | --- | --- | --- | --- | --- |
| Biometric features |  |  |  |  |  |
| Female age (years)   - Whole group - Questionnaires - Diary | 92  81  44 | 33.8 (4.2)  33.8 (4.2)  33.8 (4.0) | 86  80  35 | 34.4 (4.0)  34.3 (3.9)  33.3 (4.7 | 0.33  0.19  0.49 |
| Body-Mass Index   - Whole group - Questionnaires - Diary | 92  81  44 | 24.4 (4.6)  24.5 (4.7)  23.9 (4.1) | 86  80  35 | 23.2 (4.4)  23.1 (4.6)  22.5 (2.7) | 0.54  0.58  0.09 |
|  |  |  |  |  |  |
| Fertility history |  |  |  |  |  |
| Duration of subfertility months   - Whole group - Questionnaires - Diary | 92  81  44 | 22.0 (16.0-28.8)  21.0 (16.0-28.8)  22.0 (16.0-27.8) | 86  80  35 | 21.0 (15.8-30.0)  21.0 (16.0-29.8)  19.0 (14.0-23.0) | 0.56  0.96  0.27 |
| Primary subfertility   - Whole group - Questionnaires - Dairy | 92  81  44 | 64 (70%)  55 (65%)  30 (68%) | 86  80  35 | 57 (66%)  52 (65%)  24 (69%) | 0.64  0.61  0.97 |

Data are mean (SD), n (%), or median (IQR).
IUI-OS = intrauterine insemination with ovarian stimulation.

Supplementary table 1. Baseline characteristics
