# Supplementary material for: The impact of expectant management compared with intrauterine insemination with ovarian stimulation on quality of life and coital frequency in couples with unexplained subfertility
Source: F S Rep. 2025 Jun 11;6(3):374–80. doi: 10.1016/j.xfre.2025.06.001 (PMC12496428; doi:10.1016/j.xfre.2025.06.001)
Supplement: Supplementary Table 2 [file mmc6.docx]

|  | Filled in at least one month of a diary | | Mean score (SD)  at baseline | Mean difference (95%CI) |
| --- | --- | --- | --- | --- |
| FertiQol | | | | |
| Relational domain | Yes | N = 67 | 79.6 | -0.21 (-4.01 to 3.59) |
|  | No | N = 84 | 79.8 |  |
| Social domain | Yes | N= 69 | 73.3 | -5.02 (-10.02 to -0.03) |
|  | No | N = 85 | 78.3 |  |
| HADS | | | | |
| Anxiety scale | Yes | N = 69 | 4.9 | 0.008 (-1.06 to 1.07) |
|  | No | N = 87 | 4.9 |  |
| Depression scale | Yes | N = 69 | 2.3 | -0.42 (-1.21 to 0.36) |
|  | No | N = 87 | 2.7 |  |

The FertiQol subscale scores were transformed into a 0–100 scale, with higher scores mean higher quality of life.
The HADS subscale scores indicate a total score, with higher scores (above 8) indicate a severe situation for anxiety or depression.

Supplementary table 2. FertiQol and HADS scores analysed based on whether women kept a diary yes or no.
